# Parvalbumin-Positive Interneurons in the Medial Prefrontal Cortex Regulate Stress-Induced Fear Extinction Impairments in Male and Female Rats

**DOI:** 10.1523/JNEUROSCI.1442-22.2023

**Published:** 2023-05-31

**Authors:** Annalise N. Binette, Jianfeng Liu, Hugo Bayer, Kennedi L. Crayton, Laila Melissari, Samantha O. Sweck, Stephen Maren

**Affiliations:** Department of Psychological & Brain Sciences, and Institute for Neuroscience, Texas A&M University, College Station, Texas 77843-3474

**Keywords:** fear extinction, parvalbumin, prefrontal cortex, rat, sex, stress

## Abstract

Stress has profound effects on fear extinction, a form of learning that is essential to behavioral therapies for trauma-related and stressor-related disorders. Recent work reveals that acute footshock stress reduces medial prefrontal cortex (mPFC) activity that is critical for extinction learning. Reductions in mPFC activity may be mediated by parvalbumin (PV)-containing interneurons via feedforward inhibition imposed by amygdala afferents. To test this hypothesis, footshock stress-induced Fos expression was characterized in PV^+^ and PV^–^ neurons in the prelimbic (PL) and infralimbic (IL) cortices. Footshock stress increased the proportion of PV^+^ cells expressing Fos in both male and female rats; this effect was more pronounced in IL compared with PL. To determine whether PV^+^ interneurons in the mPFC mediate stress-induced extinction impairments, we chemogenetically silenced these neurons before an immediate extinction procedure in PV-Cre rats. Clozapine-*N*-oxide (CNO) did not affect conditioned freezing during the extinction procedure. However, CNO exacerbated extinction retrieval in both male and female rats with relatively high PL expression of designer receptors exclusively activated by designer drugs (DREADD). In contrast, in rats with relatively high IL DREADD expression, CNO produced a modest facilitation of extinction in the earliest retrieval trials, but in male rats only. Conversely, excitation of IL PV interneurons was sufficient to impair delayed extinction in both male and female rats. Finally, chemogenetic inhibition of IL-projecting amygdala neurons reduced the immediate extinction deficit in male, but not female rats. These results reveal that PV interneurons regulate extinction learning under stress in a sex-dependent manner, and this effect is mediated by amygdaloprefrontal projections.

**SIGNIFICANCE STATEMENT** Stress significantly impairs the memory of fear extinction, a type of learning that is central to behavioral therapies for trauma-based and anxiety-based disorders (e.g., post-traumatic stress disorder). Here we show that acute footshock stress recruits parvalbumin (PV) interneurons in the medial prefrontal cortex (mPFC) of male and female rats. Silencing mPFC PV interneurons or mPFC-projecting amygdala neurons during immediate extinction influenced extinction retrieval in a sex-dependent manner. This work highlights the role for PV-containing mPFC interneurons in stress-induced impairments in extinction learning.

## Introduction

Fear memory consolidation in the immediate aftermath of threat exposure is adaptive and necessary for future threat survival. One factor that may immunize fear memory to disruption is the deleterious effect that stress has on extinction learning (Maren and Holmes, 2016). Although maintaining the durability of fear memory is adaptive, stress-induced extinction impairments undermine behavioral therapies for trauma-related and anxiety-related disorders in humans (e.g., exposure therapy). For example, after acute stress, long-term extinction memory is compromised and the likelihood of fear relapse is increased (Izquierdo et al., 2006; Chauveau et al., 2012; Knox et al., 2012; Maroun et al., 2013). Moreover, when extinction training is performed shortly after fear conditioning, animals fail to encode long-term extinction memories. This stress-induced extinction impairment is termed the “immediate extinction deficit” (IED; Maren and Chang, 2006; Chang and Maren, 2009). Here we use the IED to model and elucidate the neural mechanisms underlying stress-induced extinction deficits.

The medial prefrontal cortex (mPFC) of rodents is composed of the prelimbic (PL) and infralimbic (IL) subdivisions, the latter of which is critical for extinction memory. Inactivation of the IL, but not the PL, during extinction training has no effect on extinction acquisition but disrupts extinction memory (Laurent and Westbrook, 2009; Sierra-Mercado et al., 2011; Do-Monte et al., 2015). Conversely, electrical stimulation of the IL inhibits conditioned fear expression (Milad et al., 2004) and optogenetic stimulation of IL glutamatergic neurons during delayed extinction training facilitates acquisition and extinction retrieval (Do-Monte et al., 2015). Likewise, successful retrieval of extinction memory is associated with increases in IL activity (Milad and Quirk, 2002; Chang et al., 2010; Wilber et al., 2011; Giustino et al., 2019). These studies indicate that under typical, low-stress conditions, IL activity drives the acquisition and expression of extinction learning.

Numerous studies indicate that stress incurs substantial medial prefrontal dysfunction (Arnsten, 2009). After acute stress, for example, footshock causes a transient increase in PL neuronal activity, but a sustained suppression in IL activity (Fitzgerald et al., 2015). Several lines of evidence suggest that dampened IL activity is sustained during immediate extinction, which is associated with reduced IL burst firing and reduced mPFC Fos expression relative to delayed-extinction controls (Chang et al., 2010; Kim et al., 2010). Mitigating dampened IL activity with electrical or pharmacological stimulation of the IL during immediate extinction is sufficient to reduce fear expression during subsequent testing (Kim et al., 2010; Chang and Maren, 2011).

The mPFC receives dense glutamatergic projections from the amygdala relaying extinction-relevant and stress-relevant signals (Senn et al., 2014), but it is unclear how stress produces prefrontal hypoactivity and extinction learning impairment. One possibility is that stress induces noradrenergic activation of the basolateral amygdala (BLA; Giustino et al., 2017, 2020; Maren, 2022), which exerts inhibitory influence over the mPFC (Floresco and Tse, 2007; Joffe et al., 2022) through parvalbumin (PV) inhibitory interneurons (McGarry and Carter, 2016). Prefrontal PV interneurons regulate principal neuron activity, are highly stress sensitive, and are implicated in fear memory (Page and Coutellier, 2019; Cummings and Clem, 2020; Cummings et al., 2021; Woodward and Coutellier, 2021; Yang et al., 2021). Here we propose that stress recruits mPFC PV interneurons and mPFC-projecting BLA neurons to ultimately impair extinction learning (Maren, 2022). To test this hypothesis, we examined footshock stress-induced Fos expression in mPFC PV interneurons in male and female rats. Further, we chemogenetically inhibited mPFC PV interneurons before immediate extinction to determine their contribution to the IED. Additionally, we assessed the efficacy of chemogenetic inhibition in mPFC PV interneurons. We also tested whether chemogenetic excitation of PV interneurons in the IL is sufficient to impair delayed extinction. Last, we chemogenetically inhibited IL projectors in the BLA before immediate extinction and assessed their contribution to the IED.

## Materials and Methods

### Subjects

A total of 204 Long–Evans rats were used in this study. In experiment 1, Long–Evans Blue Spruce rats (*n* = 24) were obtained from Envigo and weighed 200–224 g on arrival. In experiment 2, PV-Cre rats (*n* = 85) were bred in house and were 4–4.5 months of age on behavioral testing. The PV-Cre rats [LE-Tg (Pvalb-iCre)20ttc] used in this study were obtained from the National Institute on Drug Abuse/National Institute of Mental Health Rat Resource and Research Center and were bred with commercially supplied wild-type Long–Evans rats (Envigo). The PV-Cre rats were genotyped and randomly assigned to groups within each litter. In experiments 3 (*n* = 31) and 4 (*n* = 64), Long–Evans Blue Spruce rats were obtained from Envigo and weighed 200-224 *g* on arrival. All rats were individually housed in a temperature-controlled and humidity-controlled vivarium, with a 14 h/10 h light/dark cycle and *ad libitum* access to food and water. Behavioral testing was conducted during the light phase. Rats were handled 1 min/d for 5 d before testing to acclimate them to the experimenter.

### Viral vectors and drugs

AAV9-hSyn-DIO-hM4Di-mCherry was purchased from Addgene (viral prep #44 362-AAV9). The adeno-associated virus (AAV) was diluted in sterile 1 × PBS to a titer of 8 × 10^12^ viral genomes (vg)/ml in experiment 2, and the undiluted stock titer (2.5 × 10^13^ vg/ml) was used in experiment 4. AAV1-S5E2-Gq-P2A-dTomato was purchased from Addgene (viral prep #135635-AAV1; stock titer, 2.1 × 10^13^ vg/ml) and diluted in sterile 1× PBS to a titer of 6 × 10^12^ vg/ml. CAV2-CreGFP was obtained from the Montpellier vector platform (Plateforme de Vectorologie de Montpellier; stock titer, 14.7 × 10^12^ pp/ml). CAV2-CreGFP was diluted in sterile Dulbecco's 1× PBS to 1.0 × 10^9^ pp/1.5 µl. Clozapine-*N*-oxide (CNO) was obtained from the NIH and dissolved in 2.5% DMSO in 0.9% sterile saline and injected systemically (5 mg/kg, i.p.).

### Surgeries

Rats were anesthetized with isoflurane (5% for induction, 1–2% for maintenance; flow rate, ∼0.8 L/min) and secured in a stereotaxic apparatus (Kopf Instruments). Ophthalmic ointment was applied to the eyes, and the scalp fur was trimmed. The scalp was disinfected with three alternating washes of 70% ethanol and povidone-iodine, incised, and retracted to expose the skull. The skull was leveled by positioning bregma and λ in the same horizontal plane (± 0.10 mm). Bilateral craniotomies were drilled above the target regions. Virus was infused using stainless steel injectors (26 gauge) connected to polyethylene tubing (catalog #PE-20, Brain Tree Scientific) and 10 µl Hamilton syringes, which were secured to a microinfusion pump (KD Scientific). The IL was infused bilaterally with 0.5 µl of AAV9-hSyn-DIO-hM4Di-mCherry (experiment 2), 0.5 µl of AA1-S5E2-Gq-dTomato (experiment 3), or 1.5 µl of CAV2-CreGFP (experiment 4) at a rate of 0.1 µl/min with the following coordinates (relative to bregma): anteroposterior (AP), +2.70; mediolateral (ML), ±3.08; dorsoventral (DV), −4.60 (30° angle). The BLA was infused with 1.0 µl of AAV9-hSyn-DIO-hM4Di-mCherry at a rate of 0.1 µl/min with the following coordinates (relative to bregma): AP, −2.9; ML, ±5.0; DV, −8.55 (experiment 4). Injectors were left at the target coordinate for 5 min after infusion to allow for diffusion. In experiment 3, several modifications were made to reduce dorsal diffusion of the virus. Before infusion, the injector was positioned 0.1 mm past the target for 5 min (to create an infusion pocket); during infusion, the injector was raised 0.1 mm every minute; after infusion, the injector rested for 10 min before withdrawing. The incision was closed with sutures, and carprofen (2 mg tablet; Bio-Serv) was administered immediately after surgery. Rats were given either 2 weeks (experiment 3) or 6 weeks (experiments 2 and 4) to recover and to allow time for sufficient viral expression before behavioral testing.

### Experimental design and statistical analysis

All behavioral procedures took place in standard rodent conditioning chambers with two aluminum walls, two Plexiglas walls and a Plexiglas ceiling (Med Associates). Three distinct contexts were used in this study. Context A was characterized by the following: chamber lights off, white room lights on, fans on, chamber doors open, a 1% ammonium hydroxide scent, and white transport boxes without sawdust bedding. Context B included the following: chamber lights on, red room lights on, fans off, chamber doors closed, a 3% acetic acid scent, and black transport boxes with sawdust bedding. Context C included the following: chamber lights off, red room lights on, fans off, chamber doors open, a 70% ethanol scent, and white transport boxes with sawdust bedding.

Auditory stimuli were delivered by a speaker mounted to the top corner of one wall, and scrambled footshock was delivered by a grid floor composed of stainless steel rods. The conditioned stimulus (CS) was a 10 s, 2 kHz acoustic tone, unless indicated otherwise, and the unconditioned stimulus (US) was a 2 s, 1 mA footshock. Each session began with a 3 min stimulus-free baseline (BL) period. Conditioning consisted of five CS–US deliveries with a 70 s intertrial interval (ITI) and a 1 min post-trial period, unless indicated otherwise. Extinction and extinction retrieval consisted of 45 CS-only deliveries with a 40 s ITI and a 3 min post-trial period. Load cell force transducers located underneath each chamber measured displacement of the chamber in response to motor activity; these voltages (±10 V) were acquired at 5 Hz and transformed to absolute values (scale, 0–100). A freezing bout was defined as 5 consecutive values <10 (freezing threshold, corresponding to 1 s of freezing). All freezing data were averaged across five-trial blocks. Each trial was composed of the CS and the post-CS period.

Inescapable footshock from the conditioning procedure served as the acute stressor in these experiments. This procedure decreases the spontaneous firing of IL principal cells (Fitzgerald et al., 2015) and induces an extinction deficit (Maren and Chang, 2006). It should be noted that a weaker conditioning procedure consisting of either fewer trials (Maren and Chang, 2006) or weaker footshocks (Giustino et al., 2020) does not result in an immediate extinction deficit. Here we use footshocks of sufficient intensity (five 0.5 s, 1 mA shocks) to induce an immediate extinction deficit.

Data were analyzed with conventional parametric statistics (StatView, SAS Institute, and GraphPad Prism). Repeated-measures ANOVA was used to assess main effects and interactions (α = 0.05). For *post hoc* analyses, Fisher's least significant difference (LSD) test was used. Results are shown as the mean ± SEM. All data are available on request.

#### Experiment 1.

In the first experiment, we examined whether footshock stress recruits mPFC parvalbumin interneurons. Male (*n* = 12) and female (*n* = 12) Long–Evans rats were randomly assigned to the following groups: Shock, No-Shock, and Home. The Shock group underwent a standard auditory tone-shock conditioning procedure, the No-Shock group underwent tone-alone exposure, and animals in the Home group remained in the home cage. Conditioning was conducted in Context A with a 10 min post-trial period. Ninety minutes after conditioning, animals were perfused, and brains were extracted for immunohistochemical processing (PV and Fos immunostaining). One male rat from the Home group was excluded because of experimenter error during immunostaining, leaving the final group sizes as follows: Home males, *n* = 3; Home females, *n* = 4; No-shock males, *n* = 4; No-shock females, *n* = 4; Shock males, *n* = 4; Shock females, *n* = 4. For conditioning data, a three-way repeated-measures ANOVA was used to assess main effects (time, shock, sex) and interactions. For the histological data, a three-way repeated-measures ANOVA was used to assess main effects (region, shock, sex) and interactions. Fisher's protected LSD was used to follow-up on main effect of shock and assess differences between experimental groups (Home, No-shock, Shock).

#### Experiment 2a.

In the second experiment, we examined whether mPFC parvalbumin inhibition rescues stress-impaired fear extinction (i.e., the IED). We used a chemogenetic strategy (Armbruster et al., 2007) in transgenic rats to selectively silence mPFC PV interneurons after CNO administration. PV-Cre male and female rats (*n* = 85) expressing an inhibitory DREADD in mPFC PV interneurons were systemically injected with either vehicle (VEH) or CNO (5 mg/kg, i.p.) 15 min before conditioning (a stressor). CNO at these doses does not influence either neuronal activity in the mPFC (Marek et al., 2018) or induce nonspecific behavioral effects (Ramanathan et al., 2018). After conditioning in Context A, animals remained in transport boxes for 15 min, then underwent immediate extinction in Context B. Animals were brought back to Context B chambers 48 h later and tested for extinction memory (i.e., retrieval). After testing, DREADD expression was assessed. Two animals were excluded because of experimenter error during tissue processing, leaving the final group sizes as follows: VEH males, *n* = 22; VEH females, *n* = 23; CNO males, *n* = 19; CNO females, *n* = 19. A three-way repeated-measures ANOVA was used to assess main effects (time, sex, drug) and interactions. Substantial viral expression outside of the target IL region was observed, along the injection track and into the PL. To gauge the effect of IL expression on these results, mCherry^+^ cells were quantified in the mPFC and the following ratio was computed: (IL mCherry^+^ cells)/(PL mCherry^+^ cells + IL mCherry^+^ cells). We then performed a median split to produce Low IL expression (ratio < 0.32) and High IL expression groups. Because of the variation in BL freezing, we also assessed the BL-subtracted data for the first four blocks of retrieval, before re-extinction occurs. A four-way repeated-measures ANOVA was used to assess main effects (time, sex, drug, region) and interactions.

#### Experiment 2b.

Next, we examined whether chemogenetic inhibition of mPFC PV interneurons reduces shock-induced Fos expression in those neurons. A subset of rats (*n* = 28) from experiment 2a were injected with VEH or CNO (5 mg/kg, i.p.) before tone-footshock conditioning in Context C, with a white noise CS. Animals remained in the chamber for 10 min after the last trial. Ninety minutes after conditioning, animals were perfused, and brains were extracted for immunohistochemical processing. One rat was excluded because of experimenter error during tissue processing, and two rats were excluded because of low viral expression (<10 mCherry^+^ cells total in the mPFC), leaving the final group sizes as follows: VEH males, *n* = 6; VEH females, *n* = 6; CNO males, *n* = 7; CNO females, *n* = 6. For freezing analysis, a three-way repeated-measures ANOVA was used to assess main effects (time, drug, sex) and interactions. For analysis of mCherry and Fos expression, a three-way repeated-measures ANOVA was used to assess main effects (region, drug, sex) and interactions.

#### Experiment 3.

In this experiment we examined whether chemogenetic excitation of PV interneurons could induce an extinction deficit in a delayed extinction procedure. Wild-type rats (*n* = 31) were injected with a viral vector (AAV-S5E2-Gq-dTomato) in the IL to drive expression of an excitatory DREADD in PV interneurons (Vormstein-Schneider et al., 2020). Animals were conditioned in Context A. The next day, animals were injected with either VEH or CNO (5 mg/kg, i.p.) 30 min before extinction in Context B. Extinction retrieval in Context B was tested 24 h later. After testing, DREADD expression was assessed. Rats in the CNO group without expression were excluded (*n* = 7), leaving the final group sizes as follows: VEH males, *n* = 6; VEH females, *n* = 9; CNO males, *n* = 5; CNO females *n* = 4). A three-way repeated-measures ANOVA was used to assess main effects (time, drug, sex) and interactions. For retrieval, the BL-subtracted freezing data in blocks 1–4 were examined.

#### Experiment 4.

In this experiment, we examined whether BLA neurons projecting to the IL contribute to the IED. To this end, the IL was injected with a retrograde canine adenovirus (CAV) expressing Cre-recombinase (CAV2-Cre), and the BLA was injected with an AAV expressing a Cre-dependent inhibitory DREADD. We used the same procedure described in experiment 2a, in which animals received systemic injections of either VEH or CNO (5 mg/kg, i.p.) 15 min before conditioning; immediate extinction and retrieval were tested 15 min and 48 h later, respectively. Upon initial testing, all animals displayed a weak IED (indicated by low levels of freezing during retrieval; data not shown). We reconditioned the animals with identical procedures, except for the use of a novel white noise CS and a novel context (Context C used throughout). Three rats died during surgery, five animals had BLA damage in the region of the viral infusions, and five animals did not exhibit viral expression. These animals were excluded from the statistical analysis leaving the final group sizes as follows: VEH males, *n* = 10; VEH females, *n* = 15; CNO males, *n* = 13; CNO females, *n* = 13. A three-way repeated-measures ANOVA was used to assess main effects (time, drug, sex) and interactions. For retrieval, we examined the BL-subtracted freezing data across the session.

### Histology

All rats were overdosed with sodium pentobarbital (100 mg/kg; Fatal-Plus, Vortech Pharmaceuticals) and transcardially perfused with 1× PBS followed by 10% formalin solution. Brains were extracted and postfixed in 10% formalin solution for ∼12 h, then stored in 30% sucrose solution for a minimum of 72 h. The brains were embedded in OCT (optimal cutting temperature) compound, frozen on dry ice and coronal sections (30 µm thick) were collected using a cryostat (−20°C; Leica Microsystems). Fluorescent images were taken at 10× magnification using a Zeiss microscope and Axio Imager software (Zen Pro 2012; see Fig. 2, schematic representations of viral expression in each experiment). For each rat, the coronal section with maximal viral spread was overlaid on the corresponding stereotaxic atlas template (Swanson, 2004). The region on each section containing labeled cells was traced and shaded at either 5% (experiment 2) or 10% opacity (experiments 3, 4). The shaded region for each rat represented the maximal area of reporter expression, not the density of reporter^+^ cells. Viral expression was based on native fluorescence for experiments 2 and 3, and on immunostained tissue for experiment 4.

For immunostaining, tissue was placed into mesh well inserts on a plate shaker at room temperature unless stated otherwise. After immunostaining, tissue sections were wet mounted on gel-subbed slides and coverslipped with Fluoromount mounting medium (Thermo Fisher Scientific). Cells positive for PV, Fos, and/or mCherry were quantified using ImageJ software (National Institutes of Health).

In experiment 1, mPFC tissue was immunostained for PV and Fos. In experiments 2a and 2b, native expression of mCherry was sufficient to visualize DREADD expression. For experiment 2b, mPFC tissue was immunostained for Fos. For PV and/or Fos immunostaining, tissue was rinsed in 1× PBS (10 min) followed by rinses in 1× PBST (PBS with 0.1% Triton-X, pH 7.4; 3 × 10 min). Tissue was then placed in PBST with 10% normal donkey serum (NDS) for 2 h and rinsed again in 1× PBST (3 × 10 min). Tissue was incubated in the primary antibody [mouse anti-parvalbumin (1:400, Sigma-Aldrich, no. P3088); guinea pig anti-c-Fos (1:500; catalog #226005, Synaptic Systems)] in PBST for 16 h at 4°C. Tissue was washed in PBST (3 × 10 min), then incubated in the secondary antibody [Alexa Fluor 488 donkey anti-mouse (1:500; catalog #715–545-151, Jackson ImmunoResearch); and biotinylated anti-guinea pig IgG (1:250; catalog #706–065-148, Jackson ImmunoResearch)] in PBST with 2% NDS for 2 h. For Fos staining, tissue was washed in PBST (3 × 10 min) then incubated in the tertiary antibody (1:200; streptavidin-conjugated Alexa Fluor 350; catalog #S11249, Thermo Fisher Scientific) in PBST with 2% NDS for 90 min. Tissue was rinsed with 1× PBS (3 × 10 min). In experiment 1, PV^+^ cells were pseudocolored red for better visualization against Fos^+^ (blue) cells.

In experiment 4, BLA tissue was immunostained for mCherry. Tissue was rinsed in 1× PBST (PBS with 0.1% Triton-X, pH 7.4; 3 × 10 min), then placed in PBST with 10% NDS for 1 h. Tissue was incubated in the primary antibody (1:500; rabbit anti-RFP; catalog #600–401-379, Rockland) in PBST for 24 h at 4°C. Tissue was rinsed in PBST (3 × 10 min) and then incubated in the secondary antibody (1:500; Cy3 donkey anti-rabbit; catalog #711–165-152, Jackson ImmunoResearch) in PBST with 1% NDS for 2 h. Tissue underwent a final rinse in PBS (3 × 10 min).

## Results

### Experiment 1: footshock stress recruits mPFC PV^+^ interneurons

**Figure 1.**
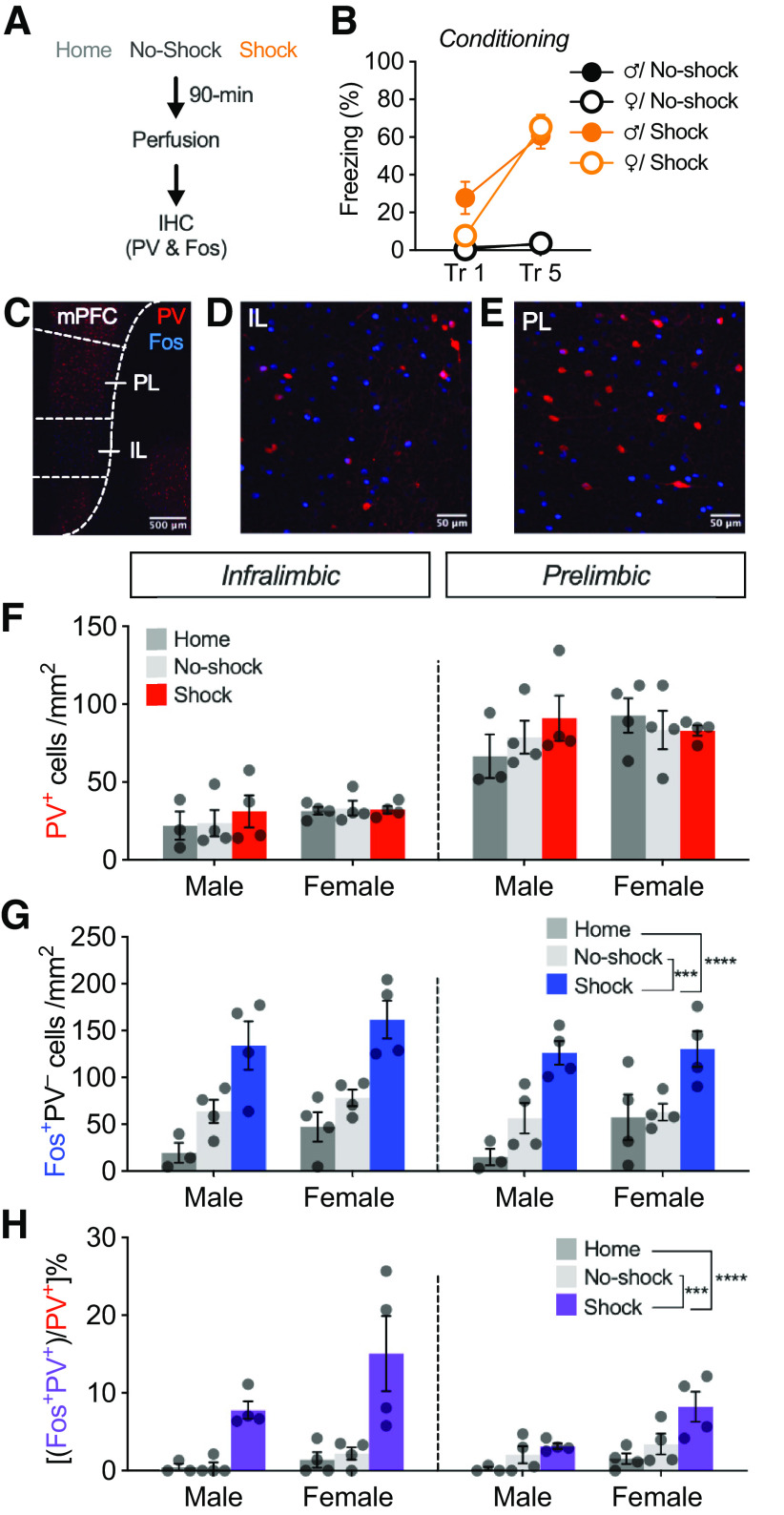
Footshock stress during fear conditioning induces Fos expression in mPFC PV interneurons in male and female rats. ***A***, Experimental design for assessing footshock stress-induced activation of mPFC PV interneurons (experiment 1). Animals underwent 5 trials of tone-shock conditioning (Shock; males, *n* = 4; females, *n* = 4), tone-only exposure (No-shock; males, *n* = 4; females, *n* = 4), or remained in the home cage (Home; males, *n* = 3; females, *n* = 4). Ninety minutes after the session, animals were perfused and brains were removed for immunohistochemistry (IHC; PV and Fos). ***B***, Percentage of freezing (mean ± SEM) during the first (Tr 1) and last (Tr 5) conditioning trials. Fear conditioning increased conditioned freezing compared with No-shock controls. ***C***, Representative image (10×) of a coronal section of the mPFC showing PV and Fos staining in a shocked male. ***D***, ***E***, Outlines depict the IL (***D***) and PL (***E***) subdivisions of the mPFC. ***F***, Density of PV^+^ cells (mean ± SEM) plotted by region, sex, and experimental group. ***G***, Density of Fos^+^ PV^–^ cells (mean ± SEM) plotted by region, sex, and experimental group. ***H***, Percentage of colabeled PV^+^ cells (mean ± SEM) plotted by region, sex, and experimental group. ***p < 0.001, ****p < 0.0001.

As a first step toward testing whether mPFC PV interneurons are involved in stress-induced extinction impairment, we assessed mPFC PV and Fos expression in response to acute inescapable footshock (Fig. 1*A*, experimental design). Animals were placed in a novel context and underwent five tone-shock conditioning trials (Shock), five tone-alone trials (No-Shock), or remained in the home cage (Home). Animals remained in the chamber for 10 min after the last trial and were perfused 90 min later. Footshock produced increases in conditioned freezing across the session relative to No-shock controls, with no differences between sex (Fig. 1*B*; main effect of shock: *F*_(1,12)_ = 359.0, *p* < 0.0001; main effect of sex: *F*_(1,12)_ = 3.725, *p* = 0.0776; shock × sex interaction: *F*_(1,12)_ = 3.327, *p* = 0.0932; time × shock × sex interaction: *F*_(1,12)_ = 1.703, *p* = 0.2164).

After footshock and perfusion, mPFC tissue was immunostained for PV and Fos (Fig. 1*C–E*). Expression of PV and Fos was quantified in the IL and PL cortices of male and female rats (Fig. 1*F–H*). First, we assessed PV expression across the IL and PL. As shown in Figure 1*F*, the PL showed a greater density of PV^+^ cells relative to the IL (main effect of region: *F*_(1,17)_ = 241.349, *p* < 0.0001). As expected, there was no effect of shock on PV^+^ cell density (*F*_(2,34)_ = 0.288, *p* = 0.7537). Additionally, there was no sex difference in PV^+^ cell density (no main effect of sex: *F*_(1,17)_ = 1.091, *p* = 0.3110). These data demonstrate that parvalbumin expression is greater in the PL cortex relative to the IL cortex in male and female rats.

Next, we examined stress-induced Fos expression in PV^–^ cells in the IL and PL cortices (Fig. 1*G*). Footshock increased the overall Fos^+^ PV^–^ cell density in the mPFC (main effect of shock: *F*_(2,34)_ = 21.808, *p* < 0.0001). There was no difference in Fos^+^ PV^–^ cell density between the IL and PL (main effect of region: *F*_(1,17)_ = 3.912, *p* = 0.0644; region × shock interaction: *F*_(2,34)_ = 1.892, *p* = 0.1812) or between sex (main effect of sex: *F*_(1,17)_ = 2.457, *p* = 0.1355; shock × sex interaction: *F*_(2,34)_ = 0.314, *p* = 0.7344; region × shock × sex interaction: *F*_(2,34)_ = 1.420, *p* = 0.2690). These data demonstrate that stress induces Fos expression in PV^–^ cells comparably across the IL and PL cortices of male and female rats.

Last, we examined Fos expression in PV^+^ cells in the IL and PL cortices (Fig. 1*H*). Footshock increased the percent of colabeled PV^+^ cells (main effect of shock: *F*_(2,34)_ = 14.625, *p* = 0.0002) above No-shock and Home controls (*post hoc*: Home vs Shock, *p* = 0.0001; No-shock vs Shock, *p* = 0.0004). Interestingly, there was significant region × shock interaction (*F*_(2,34)_ = 9.238, *p* = 0.0019), with the shock group showing a greater proportion of colabeled PV^+^ cells in the IL relative to the PL (*post hoc*: Shock group IL vs PL, *p* = 0.0150). Although there was a main effect of sex (*F*_(1,17)_ = 5.458, *p* = 0.0320), there was no shock × sex interaction (*F*_(2,34)_ = 1.691, *p* = 0.2138), region × sex interaction (*F*_(1,17)_ = 0.250, *p* = 0.6238), or region × shock × sex interaction (*F*_(2,34)_ = 0.276, *p* = 0.7624). Overall, these data indicate that footshock stress recruits mPFC PV interneurons similarly in male and female rats, to a greater degree in the IL cortex relative to the PL cortex.

### Experiment 2a: chemogenetic inhibition of PV^+^ cells in the mPFC mediates stress-impaired extinction in male and female rats

**Figure 2.**
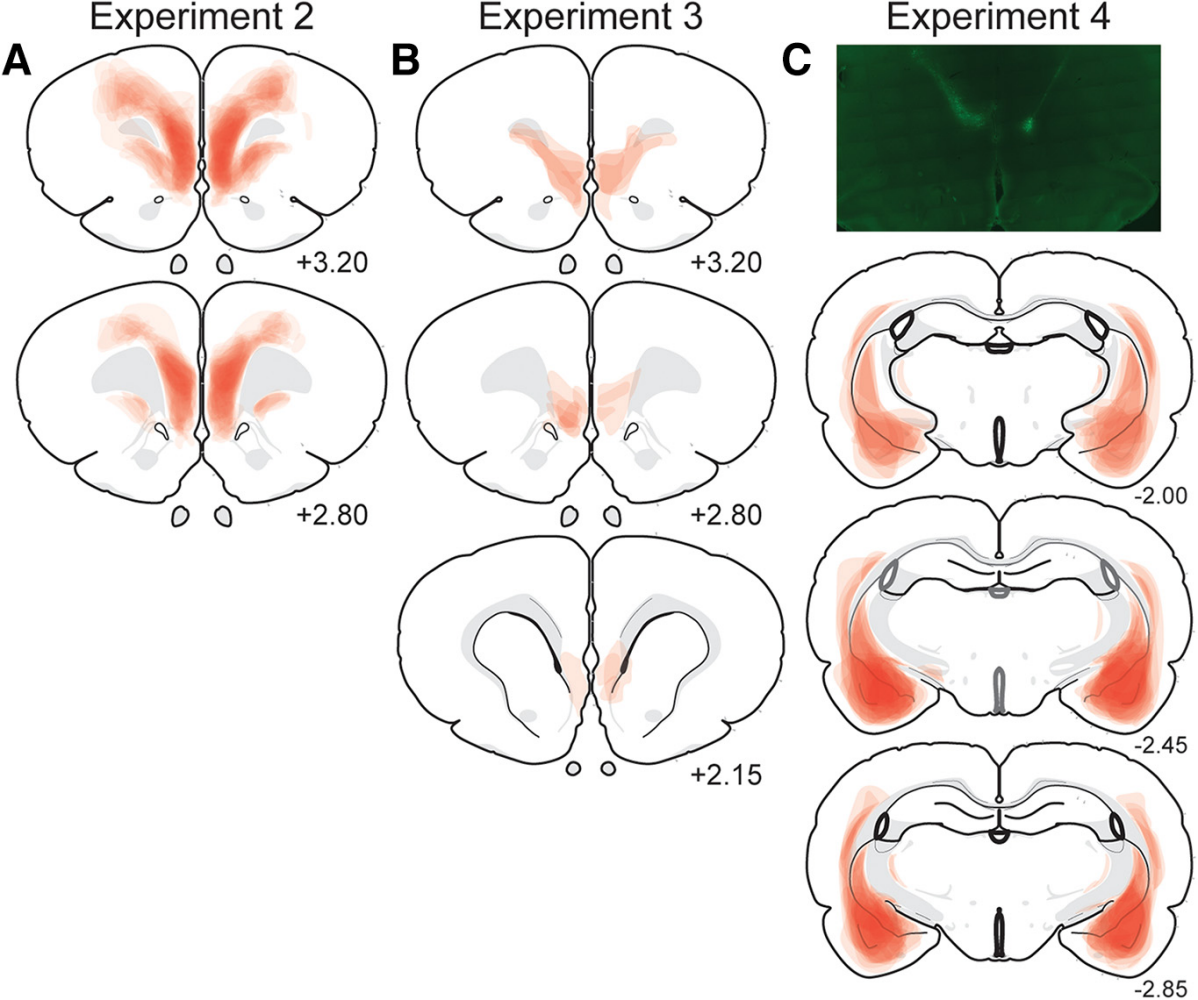
Schematic representation of viral expression for all of the animals in each experiment (viral expression common to all rats is indicated by the most darkly shaded areas). ***A***, Maximal viral spread of DIO-hM4Di-mCherry in the mPFC for each subject (experiment 2). ***B***, Maximal viral spread of S5E2-Gq-dTomato in the IL for each subject (experiment 3). ***C***, Representative immunofluorescent image of CAV2-Cre-GFP in the IL (top panel) and maximal viral spread of DIO-hM4Di-mCherry in the BLA for each subject (bottom panels; experiment 4).

**Figure 3.**
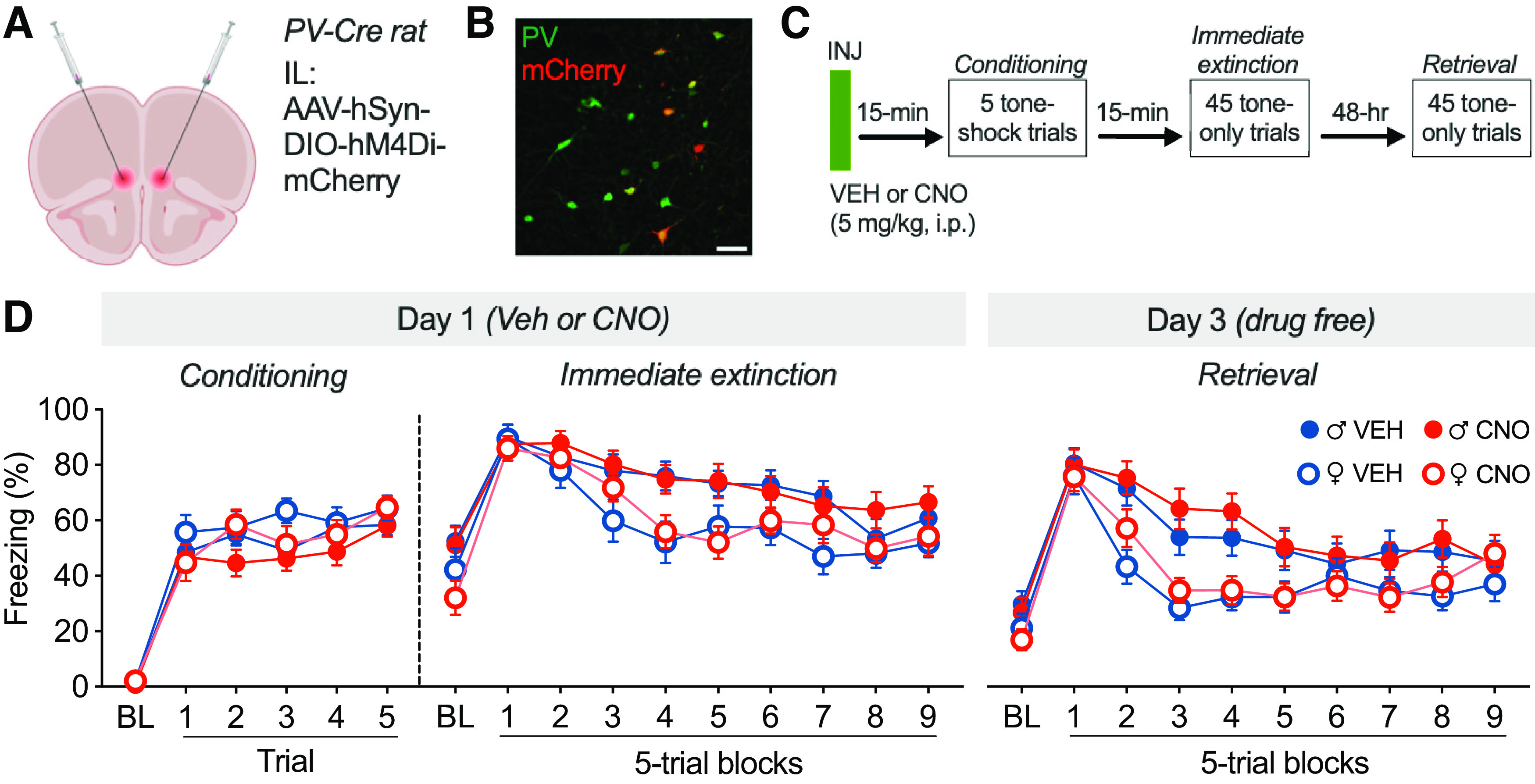
Chemogenetic inhibition of mPFC PV interneurons before immediate extinction in male and female rats. ***A***, Viral strategy for expressing hM4Di selectively in mPFC PV interneurons of PV-Cre transgenic rats. ***B***, PV-immunostained tissue of the mPFC showing colabeling of PV and mCherry expression (the rightmost mCherry-expressing cell also colocalized with a faint green signal not visible in the image). Scale bar, 50 µm. ***C***, Experimental design for assessing the contribution of mPFC PV interneurons to the IED (experiment 2a). Animals were injected with VEH or CNO before conditioning (a stressor) and immediate extinction, then were tested for extinction retrieval 48 h later. ***D***, Percentage of freezing (mean ± SEM) across conditioning, immediate extinction, and retrieval, plotted by sex and drug group [VEH males (♂), *n* = 22; CNO ♂, *n* = 23; VEH females (♀), *n* = 19; ♀ CNO, *n* = 19]. Treatment with CNO resulted in nonsignificant increases in freezing during retrieval. Elements of this figure were created with bioRENDER.

Because we observed stress-induced activation of mPFC PV interneurons, we next asked whether silencing those cells could rescue stress-induced extinction impairment. Transgenic PV-Cre rats were infused with an AAV encoding a Cre-dependent inhibitory DREADD into the IL (Figs. 2*A*, 3*A*). Expression of hM4Di-mCherry was concentrated in the mPFC, with some diffusion into the anterior cingulate, claustrum, and motor areas (Fig. 2*A*). To determine the specificity of DREADD expression in the mPFC, we examined colocalization of hM4Di-mCherry expression in tissue immunostained for PV (*n* = 3 rats). As shown in Figure 3*B*, hM4Di-mCherry is expressed in neurons that also expressed PV. We observed that 74.3 ± 8.9% of mCherry^+^ cells expressed PV and 28.5 ± 11.7% of PV^+^ cells expressed mCherry. These data suggest that although DREADD expression is selective for PV, expression is restricted to about one-fourth of PV cells.

To assess the role of PV interneurons in the IED, rats were injected with VEH or CNO (5 mg/kg, i.p.) before conditioning (a stressor), which preceded an immediate extinction session by 15 min; extinction memory was tested 48 h later (Fig. 3*C*). We administered CNO before conditioning, because increases in mPFC and BLA firing occur immediately after footshock (Fitzgerald et al., 2015; Giustino et al., 2020). As shown in Figure 3*D*, all groups showed an increase in freezing behavior during conditioning (main effect of time: *F*_(5,395)_ = 124.6, *p* < 0.0001). There was no effect of CNO (main effect of drug: *F*_(1,79)_ = 2.421, *p* = 0.1237; time × drug interaction: *F*_(5,395)_ = 0.6970, *p* = 0.6260) and no sex differences (main effect of sex: *F*_(1,79)_ = 3.854, *p* = 0.0532, time × sex interaction: *F*_(5,395)_ = 0.7156, *p* = 0.6120; sex × drug interaction: *F*_(1,79)_ = 0.0029, *p* = 0.9574; time × sex × drug interaction: *F*_(5,395)_ = 1.060, *p* = 0.3821).

During immediate extinction (Fig. 3*D*), all groups showed a comparable peak in initial CS-evoked freezing (main effect of time: *F*_(9,711)_ = 36.88, *p* < 0.0001), with females showing a more rapid reduction in freezing relative to males (main effect of sex: *F*_(1,79)_ = 8.651, *p* = 0.0043; time × sex interaction: *F*_(9,711)_ = 1.934, *p* = 0.0445) as we have previously observed (Binette et al., 2022). There was no effect of CNO during immediate extinction (main effect of drug: *F*_(1,79)_ = 0.1529, *p* = 0.6968; time × drug interaction: *F*_(9,711)_ = 1.001, *p* = 0.4376; sex × drug interaction: *F*_(1,79)_ = 0.0050, *p* = 0.9437; time × sex × drug interaction: *F*_(9,711)_ = 0.8465, *p* = 0.5734). During extinction retrieval (Fig. 3*D*), all groups showed high levels of initial CS-evoked freezing, indicative of the IED (main effect of time: *F*_(9,711)_ = 50.74, *p* < 0.0001). Females again showed a more rapid reduction in freezing relative to males (main effect of sex: *F*_(1,79)_ = 9.418, *p* = 0.0029; time × sex interaction: *F*_(9,711)_ = 4.696, *p* < 0.0001). However, we did not observe a significant effect of CNO on the expression of conditioned freezing during the drug-free retrieval test (main effect of drug: *F*_(1,79)_ = 0.3043, *p* = 0.5827; time × drug interaction: *F*_(9,711)_ = 1.264, *p* = 0.2529; sex × drug interaction: *F*_(1,79)_ = 0.0016, *p* = 0.9680; time × sex × drug interaction: *F*_(9,711)_ = 0.6227, *p* = 0.7783).

**Figure 4.**
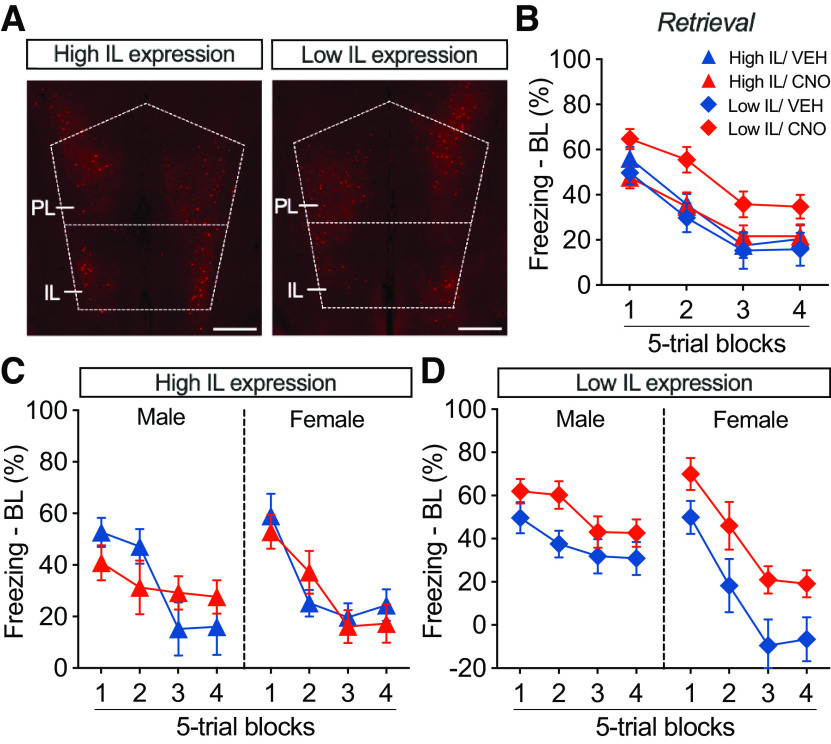
The effect of chemogenetic mPFC PV inhibition on stress-induced extinction impairment depends on sex and localization of hM4Di expression. ***A***, Representative coronal sections through the mPFC showing examples of subjects with relatively high or low IL hM4Di-mCherry expression. Scale bar, 500 µm. ***B***, Percentage of freezing (normalized to baseline; mean ± SEM) during early retrieval blocks, split by high or low IL hM4Di expression. ***C***, Percentage of freezing (normalized to baseline; mean ± SEM) during early retrieval in male and female rats with relatively high IL expression. CNO-treated males, but not females, with high IL hM4Di expression showed an initial modest reduction in freezing relative to vehicle controls. ***D***, Percentage of freezing (normalized to baseline; mean ± SEM) during early retrieval in male and female rats with relatively low IL expression. CNO-treated males and females with low IL hM4Di expression showed higher levels of freezing relative to vehicle controls.

We considered the possibility that the lack of effect of CNO on the immediate extinction deficit may have been because of individual differences in the levels and/or localization of hM4Di-mCherry expression in the IL and PL cortices. To examine potential region-specific (IL vs PL) effects, we quantified hM4Di-mCherry expression within the mPFC. We then calculated the ratio of mCherry^+^ cells in the IL relative to total mCherry^+^ cells in the mPFC (IL + PL). This ratio was then used to perform a median split and divide the animals into groups with relatively high levels of IL expression (mean = 0.47 ± 0.02) and groups with relatively lower levels of IL expression (mean = 0.18 ± 0.02). Examples of coronal sections of animals in each of these groups are shown in Figure 4*A*. Notably, despite our efforts to selectively express hM4Di in the IL, most of the expression (even in “high-IL” animals) was in the PL because of dorsal diffusion of the virus (Fig. 2*A*).

Because freezing during the retrieval session extinguished relatively rapidly (Fig. 3*D*), we focused our attention on the first four blocks of the retrieval session. To account for variability in BL freezing, we normalized freezing during the retrieval trials by subtracting the pretrial baseline. As shown in Figure 4*B*, CNO-treated animals (collapsed across sex) showed a significant impairment in retrieval relative to VEH controls (main effect of drug: *F*_(1,79)_ = 4.402, *p* = 0.0393), and this effect was most pronounced in animals with relatively low IL hM4Di expression (region × drug interaction: *F*_(1,79)_ = 5.241, *p* = 0.0249). Interestingly, this outcome was influenced by the sex of the subjects. As shown in Figure 4*C*, in rats with relatively high levels of IL hM4Di expression, CNO treatment during immediate extinction resulted in a modest reduction in conditioned freezing in the earliest trials of the retrieval session. This effect was not apparent in female rats. In contrast, in rats expressing relatively low levels of hM4Di in the IL, CNO treatment led to similar impairments in extinction retrieval in both male and female rats. These observations were confirmed in a four-way repeated-measures ANOVA that revealed a significant time × sex × drug × region interaction (*F*_(3,225)_ = 3.811, *p* = 0.0108). These results suggest that silencing of PV interneurons in the PL during immediate extinction exacerbates the IED and promotes extinction retrieval deficits, whereas silencing IL PV interneurons, at least in male rats, weakens the IED and leads to a modest increase in extinction retrieval.

### Experiment 2b: chemogenetic inhibition of mPFC PV cells reduces shock-induced Fos expression

**Figure 5.**
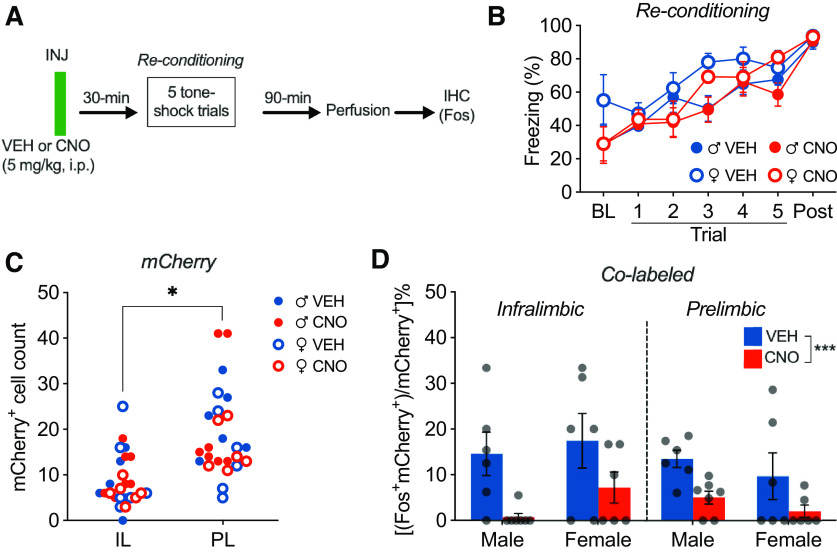
Chemogenetic inhibition of mPFC PV interneurons reduces shock-induced Fos expression in those neurons. ***A***, Experimental design for DREADD validation using immunohistochemical (IHC) Fos staining (experiment 2b). ***B***, Percentage of freezing (mean ± SEM) across the conditioning session plotted by sex and drug group [VEH males (♂), *n* = 6; CNO ♂, *n* = 7; VEH females (♀), *n* = 6; CNO ♀, *n* = 6]. Treatment with CNO before conditioning had no effect on freezing across the conditioning session. ***C***, Number of mCherry^+^ cells in the IL and PL cortex (symbols represent data from individual subjects). There was significantly greater mCherry expression in the PL compared with the IL, with no differences in expression across groups. ***D***, Percentage of colabeled mCherry^+^ (mean ± SEM) in the IL and PL, plotted by sex and drug group. Treatment with CNO before conditioning significantly reduced the proportion of mCherry^+^ cells expressing Fos. *p < 0.05, ***p < 0.001.

To gauge DREADD inhibition efficacy, we next asked whether CNO administration reduces footshock-induced Fos expression in mPFC PV interneurons in a subset of animals from experiment 2a. One week after the final retrieval test in experiment 2a, the rats were injected with VEH or CNO (5 mg/kg, i.p.) and reconditioned in a novel context with a novel CS, with perfusion 90 min later (Fig. 5*A*).

As shown in Figure 5*B*, all groups showed an increase in conditioned freezing (main effect of time: *F*_(6,126)_ = 30.51, *p* < 0.0001), with no effect of CNO (main effect of drug: *F*_(1,21)_ = 1.855, *p* = 0.1876; no time × treatment interaction: *F*_(6,126)_ = 0.8946, *p* = 0.5011). Although females showed greater levels of freezing overall (main effect of sex: *F*_(1,21)_ = 5.561, *p* = 0.0281), all groups showed comparable levels of freezing during the postshock period. As shown in Figure 5*C*, mCherry expression was greater in the PL relative to the IL (main effect of region: *F*_(1,21)_ = 46.49, *p* < 0.0001), with no differences between groups (main effect of drug: *F*_(1,21)_ = 0.0041, *p* = 0.9494; main effect of sex: *F*_(1,21)_ = 1.726, *p* = 0.2031, sex × drug interaction: *F*_(1,21)_ = 0.2862, *p* = 0.5983). As shown in Figure 5*D*, animals treated with CNO before footshock showed a reduction in the proportion of hM4Di-mCherry^+^ cells expressing Fos (main effect of drug: *F*_(1,21)_ = 19.63, *p* = 0.0002) across regions and sex (region × drug interaction: *F*_(1,21)_ = 0.5641, *p* = 0.4610; sex × drug interaction: *F*_(1,21)_ = 0.2309, *p* = 0.6358; region × sex × drug interaction: *F*_(1,21)_ = 0.0717, *p* = 0.7915). These data indicate that chemogenetic inhibition of mPFC PV interneurons before footshock is effective in reducing activity in those cells (at least as indexed by Fos expression).

### Experiment 3: chemogenetic excitation of IL PV interneurons impairs delayed extinction in male and female rats

The results from experiment 2 suggest that chemogenetic inhibition of PV interneurons in IL regulates extinction learning. However, hM4Di expression was found in both IL and PL, complicating interpretation of the data. To address this issue, we chose a complementary approach in an effort to more selectively target IL and address whether chemogenetic activation of PV interneurons in IL would precipitate extinction deficits mirroring the IED. To this end, we chemogenetically excited PV interneurons in IL before delayed extinction (24 h postconditioning).

**Figure 6.**
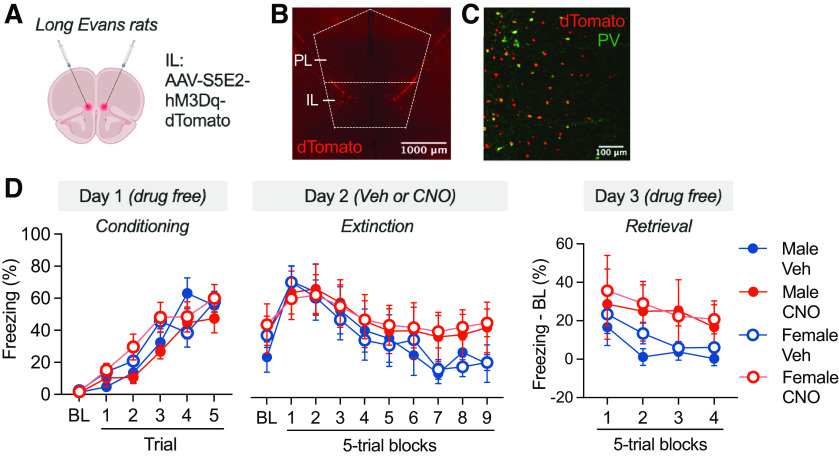
Chemogenetic excitation of IL PV interneurons before delayed extinction (under low stress). ***A***, Viral strategy for expressing hM3Dq selectively in IL PV interneurons of wild-type rats (experiment 3). ***B***, mPFC tissue showing dTomato expression in the IL. Scale bar, 500 µm. ***C***, PV-immunostained tissue of the IL showing colabeling of PV and dTomato expression in separate pilot animals. Scale bar, 100 µm. ***D***, Percentage of freezing (mean ± SEM) across conditioning, extinction, and retrieval, plotted by sex and drug group [VEH males (♂), *n* = 6; CNO ♂, *n* = 5; VEH females (♀), *n* = 9; CNO ♀, *n* = 4]. Treatment with CNO before delayed extinction resulted in impairment of extinction retrieval. Elements of this figure were created with bioRENDER.

Wild-type rats received IL infusions of an AAV encoding for an excitatory DREADD driven by a PV cell type-specific promoter (Fig. 6*A–C*; AAV-S5E2-Gq-dTomato; Vormstein-Schneider et al., 2020). Viral expression was localized to the IL with minimal dorsolateral spread into surrounding areas (Fig. 2*B*). Animals were conditioned and 24 h later received VEH or CNO (5 mg/kg, i.p.) 30 min before extinction; retrieval was tested 24 h later. As shown in Figure 6*D*, all animals showed similar increases in freezing across the conditioning session (main effect of time: *F*_(5,100)_ = 54.42, *p* < 0.0001; main effect of sex: *F*_(1,20)_ = 1.761, *p* = 0.1995), with females showing slightly higher levels of freezing toward the end of the session (time × sex interaction: *F*_(5,100)_ = 3.495, *p* = 0.0059). There was no difference in conditioning between the preassigned drug groups (main effect of drug: *F*_(1,20)_ = 0.1302, *p* = 0.7220; sex × drug interaction: *F*_(1,20)_ = 1.039, *p* = 0.3202; time × sex × drug interaction: *F*_(5,100)_ = 1.423, *p* = 0.2225).

During extinction, all groups showed initial increases in CS-evoked freezing followed by a reduction in freezing across the session (main effect of time: *F*_(9,180)_ = 10.36, *p* < 0.0001), which was not affected by sex or CNO (main effect of sex: *F*_(1,20)_ = 0.3972, *p* = 0.5357; main effect of drug: *F*_(1,20)_ = 1.900, *p* = 0.1833; time × drug interaction: *F*_(9,180)_ = 1.246, *p* = 0.2700; time × sex × drug interaction: *F*_(9,180)_ = 0.2266, *p* = 0.9904). However, during extinction retrieval, CNO-treated male and female rats exhibited higher levels of freezing (normalized to baseline) in the first four blocks of the session relative to VEH controls (main effect of drug: *F*_(1,20)_ = 4.407, *p* = 0.0487), with no sex difference (main effect of sex: *F*_(1,20)_ = 0.3824, *p* = 0.5433; sex × drug interaction: *F*_(1,20)_ = 0.0553, *p* = 0.8164; time × sex × drug interaction: *F*_(3,60)_ = 0.0835, *p* = 0.9688). Although there were differences in the quantity of virally transduced neurons in the wild-type rats used in this experiment relative to the PV-Cre rats used in experiment 2, we nonetheless observed a behavioral effect. These data suggest that IL PV excitation has no effect on extinction acquisition under relatively low-stress conditions, but impairs subsequent extinction memory in male and female rats.

### Experiment 4: chemogenetic inhibition of IL projectors in the BLA reduces the IED in male, but not female, rats

**Figure 7.**
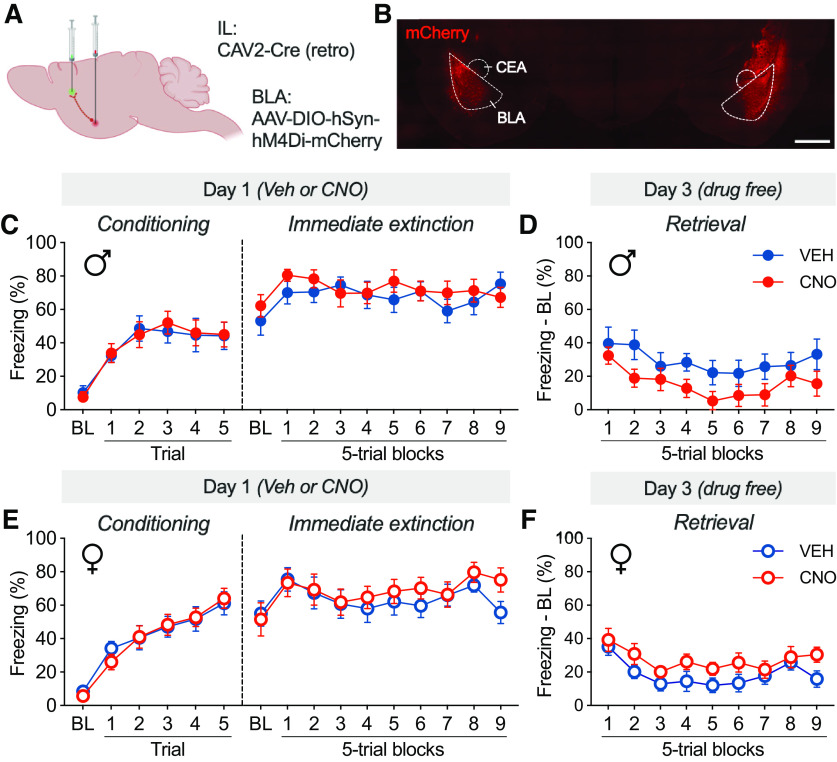
Chemogenetic inhibition of BLA neurons projecting to the IL cortex reduces stress-induced extinction impairment in male, but not female, rats. ***A***, Viral strategy for expressing hM4Di selectively in IL-projecting BLA neurons (experiment 4). ***B***, Representative histology showing hM4Di-mCherry expression in the BLA and CEA. Scale bar, 200 µm. ***C***, Percentage of freezing (mean ± SEM) across conditioning and immediate extinction in males, plotted by drug group (VEH males, *n* = 10; CNO males, *n* = 13). ***D***, Percentage of freezing with (normalized to baseline; mean ± SEM) during retrieval in males, plotted by drug group. ***E***, Percentage of freezing (mean ± SEM) across conditioning and immediate extinction in females, plotted by drug group (VEH females, *n* = 15; CNO females, *n* = 13). ***F***, Percentage of freezing (normalized to baseline; mean ± SEM) during retrieval in females, plotted by drug group. Elements of this figure were created with bioRENDER. ♂, Male; ♀, female.

Previous data from our laboratory and others provide strong evidence that stress-induced deficits arise from amygdala-mediated prefrontal inhibition (Fitzgerald et al., 2015; Giustino et al., 2017, 2019, 2020). We therefore hypothesized that silencing IL projectors in the BLA would rescue the IED. To test this hypothesis, we used an intersectional approach in which a retrograde CAV expressing Cre was injected into the IL and an AAV expressing a Cre-dependent inhibitory DREADD was injected into the BLA (Fig. 7*A*). This resulted in hM4Di-mCherry expression localized to the BLA; in IL-projecting neurons as well as some (presumably nonspecific) expression in surrounding regions of the central amygdala (CEA) and posterior striatum (Fig. 2*C*, 7*B*). Animals were injected with VEH or CNO (5 mg/kg, i.p.) before conditioning and immediate extinction, then tested for retrieval 48 h later.

As shown in Figure 7, *C* and *E*, male and female rats acquired similar levels of conditioned freezing (main effect of time: *F*_(5,235)_ = 61.64, *p* < 0.0001; main effect of sex: *F*_(1,47)_ = 0.1713, *p* = 0.6808), with females showing slightly higher levels of freezing toward the end of the session (time × sex interaction: *F*_(5,235)_ = 4.236, *p* = 0.0010). There was no effect of drug treatment on conditioning (main effect of drug: *F*_(1,47)_ = 0.0020, *p* = 0.9648). A similar pattern of results was observed during the immediate extinction procedure in which both male (Fig. 7*C*) and female (Fig. 7*E*) rats exhibited high levels of freezing that were not affected by CNO administration (main effect of time: *F*_(9,423)_ = 4.821, *p* < 0.0001; main effect of sex: *F*_(1,47)_ = 0.4475, *p* = 0.5068; main effect of drug: *F*_(1,47)_ = 0.6444, *p* = 0.4264). However, CNO administration during the immediate extinction procedure reduced freezing during the subsequent retrieval test in male, but not female, rats. Male rats treated with CNO (Fig. 7*D*) exhibited reduced CS-elicited freezing behavior (normalized to baseline) during drug-free retrieval testing, whereas female rats (Fig. 7*F*) did not. This observation was confirmed in a three-way repeated-measures ANOVA, which revealed a significant sex × drug interaction (*F*_(1,47)_ = 6.252, *p* = 0.0159). These data suggest that inhibition of the BLA–IL pathway during stress is sufficient to improve stress-induced extinction deficits in male, but not female, rats.

## Discussion

The present results reveal that acute footshock stress experienced during pavlovian fear conditioning recruits PV^+^ interneurons in the mPFC (particularly IL) in male and female rats. Inhibition of PV interneurons during fear conditioning and immediate extinction regulated the IED in a sex-dependent and region-dependent manner. In rats expressing relatively high levels of inhibitory DREADDs in the IL, male, but not female, rats exhibited a modest attenuation of the IED. In contrast, male and female rats with relatively low IL hM4Di expression (and high PL expression) exhibited an exacerbated IED. Conversely, chemogenetic excitation of IL PV interneurons during delayed extinction impaired extinction retrieval in both male and female rats. Lastly, we observed that the inhibition of IL projectors in the BLA reduced stress-induced extinction deficits in male, but not female, rats.

In experiment 1, we showed that acute inescapable footshock stress increases mPFC Fos expression in PV^+^ cells comparably in male and female rats, particularly in the infralimbic cortex. It is possible that some of the observed Fos expression is related to associative factors rather than footshock stress per se. However, these results are consistent with other work showing that unpredictable chronic mild stress increases Fos expression in mPFC PV interneurons of male and female mice (Page et al., 2019). Additionally, we observed that the density of PV^+^ cells in the mPFC are comparable among male and female rats, with a greater PV density in the PL relative to the IL. The density of mPFC PV^+^ cells was consistent across control and shock experimental groups, suggesting that acute stress itself does not alter parvalbumin expression. This is in line with work showing that acute elevated-platform stress alone has no effect on mPFC PV or GAD67 expression (Moench et al., 2020).

We also observed overall increases in shock-induced Fos expression in PV^–^ mPFC neurons. These data are in line with studies that have used other acute stressors, including swim or restraint stress. Cullinan et al. (1995) showed that both acute swim and restraint stress produces increases in mPFC Fos mRNA expression in male rats. With regard to sex, Kim and Chung (2021) showed that acute restraint stress induces Fos expression comparably in the mPFC of male and female mice, similar to the results we present here. However, Sood et al. (2018) showed that while acute immobilization stress induces Fos expression in the mPFC of both male and female rats, males showed greater stress-induced IL activation relative to females. They also observed forced swim-induced mPFC Fos expression, with females showing greater activation in the PL relative to the IL. These studies indicate that sex differences in the manifestation of stress may depend on the severity of the stressor.

The observation of shock-induced increases in mPFC Fos expression was not expected based on our prior findings of shock-induced reduction of IL activity (Fitzgerald et al., 2015; Giustino et al., 2017). However, it is important to note that the relatively broad temporal window of Fos expression limits the assessment of activity within narrow timepoints (e.g., acquisition vs postconditioning). We speculate that the increases in shock-induced IL Fos expression we have observed reflect the robust but transient post-US increase in mPFC spike firing, which rapidly decreases thereafter (Fitzgerald et al., 2015). Additionally, stress-induced Fos^+^ cells likely represent a heterogeneous population, including principal cells and a variety of interneurons alike. Future studies assessing the activity of multiple cell types with greater temporal resolution (e.g., with fiber photometry) would be instrumental in further understanding the highly dynamic effects of stress on mPFC activity.

It has been proposed that stress-induced activation of IL PV interneurons, as we have shown here, produces feedforward inhibition (Floresco and Tse, 2007; Dilgen et al., 2013; McGarry and Carter, 2016) and reduction of extinction-critical IL neuronal activity (Maren, 2022). It is well established that IL activity after extinction learning is crucial for long-term extinction memory. For example, NMDAR-dependent burst firing in the IL after extinction training is required for extinction memory retrieval (Burgos-Robles et al., 2007). In the present study, we found that mitigating stress-induced reductions in IL activity by silencing mPFC PV interneurons before conditioning and immediate extinction reduces the IED under some conditions. However, this effect was sex dependent and varied with the localization of DREADD expression in the mPFC. Other studies have also shown adaptive effects of IL PV inhibition during acute stress. For example, IL PV inhibition drives passive coping responses during a tail suspension test (Nawreen et al., 2020).

There are other caveats to consider when interpreting the results of our chemogenetic PV mPFC manipulation. The first is that viral expression extended beyond the intended target region of the IL, and into the PL and other cortical regions, including portions of the claustrum, orbitofrontal cortex, and motor areas. We cannot exclude the possibility that PV inhibition in these areas contributed to our results. For example, the inhibition of PV interneurons in PL would have led to a disinhibition of principal neurons in PL and contributed to the increases in fear expression that we observed (Courtin et al., 2014). It is possible that off-target effects of CNO may have influenced the outcomes we observed, although two points argue against this possibility. First, we have previously shown that CNO does not produce nonspecific locomotor effects at the doses used in the present work (Ramanathan et al., 2018) and does not affect activity in mPFC (Giustino et al., 2019). Second, we observed no effects of CNO on the expression of conditioned freezing when the animals were tested on drug; behavioral differences only emerged during drug-free extinction retrieval tests.

Interestingly, we observed significant sex differences on both the effects of DREADDs on extinction retrieval as well as conditioned freezing during extinction retrieval in control animals. In control rats, females displayed an IED that was comparable to males, but subsequently re-extinguished more rapidly than males, a pattern we have previously observed (Binette et al., 2022). In CNO-treated animals with high IL hM4Di expression, males, but not females, showed reduced freezing during early retrieval. In contrast to the sex-dependent effect of IL PV inhibition, IL PV excitation during delayed extinction yielded an extinction deficit in both male and female rats. It is unclear which neural mechanisms underlie these basal sex differences and their regulation. In males, BLA-evoked excitation is stronger in IL PV interneurons relative to BLA-projecting IL principal neurons and somatostatin interneurons (McGarry and Carter, 2016). One possibility is that there are sex differences in the strength of BLA input to IL PV interneurons. Additionally, recent evidence suggests that the estrus cycle robustly alters cortical PV firing. Clemens et al. (2019) showed that estradiol increases PV excitability via estrogen receptor β (Erβ), and that Erβ expression in PV neurons is increased during proestrus. In our study, increased proestrus-driven and estradiol-driven excitability of PV neurons might partially explain the lack of behavioral effect of inhibition in females.

Previous work from our laboratory has shown that systemic antagonism of β-noradrenergic receptors stabilizes shock-induced changes in mPFC activity (Fitzgerald et al., 2015). Moreover, intra-BLA, but not intra-mPFC, noradrenergic blockade rescues the IED (Giustino et al., 2017). We therefore hypothesized that stress-induced increases in noradrenaline and BLA activity might contribute to IL dysregulation and extinction impairment (Bierwirth and Stockhorst, 2022; Maren, 2022). We found that circuit-specific inhibition of IL projectors in the BLA reduced the IED in male, but not female, rats. One possibility is that there are sex differences in amygdala–prefrontal circuits, which are required for fear extinction (Senn et al., 2014; Bukalo et al., 2015; Bloodgood et al., 2018). For example, extinction resistant male, but not female, rats have reduced dendritic length in BLA-projecting IL neurons (Gruene et al., 2015), whereas in ovariectomized female rats, estrogen treatment in combination with stress induces dendritic expansion in BLA-projecting IL neurons (Shansky et al., 2010). This could result in less top-down BLA inhibition in males relative to females, and greater sensitivity of the IED to BLA manipulations in male rats. Future studies could unravel potential circuit-level sex differences by comparing stress-induced Fos expression in BLA → IL, locus coeruleus (LC) → BLA and LC → IL neurons (and noradrenergic receptor expression within these circuits) between males and females.

In conclusion, the present study demonstrates the sex-dependent influence of acute stress on amygdala–prefrontal inhibitory circuity and extinction memory. We show that footshock stress recruits mPFC PV interneurons and that IL PV interneurons bidirectionally modulate extinction; excitation of IL PV interneurons induces an extinction deficit in both sexes, while inhibition of IL PV interneurons before immediate extinction reduces extinction deficits in males, but not females. Similarly, inhibition of IL-projecting BLA neurons before immediate extinction reduces extinction deficits in male rats. These findings support a broader model in which stress induces noradrenergic activation of the BLA, resulting in feedforward inhibition of mPFC principal cells to ultimately dysregulate extinction-critical neuronal activity. Overall, these data give insight into the sex-specific behavioral and circuit-level alterations induced by stress. Importantly, these data may also reveal neurobiological mechanisms underlying poor long-term efficacy of extinction-based psychotherapy in patients with trauma-related and stressor-related disorders.

## References

[B1] Armbruster BN, Li X, Pausch MH, Herlitze S, Roth BL (2007) Evolving the lock to fit the key to create a family of G protein-coupled receptors potently activated by an inert ligand. Proc Natl Acad Sci U S A 104:5163–5168. 10.1073/pnas.0700293104 17360345 PMC1829280

[B2] Arnsten AFT (2009) Stress signalling pathways that impair prefrontal cortex structure and function. Nat Rev Neurosci 10:410–422. 10.1038/nrn2648 19455173 PMC2907136

[B3] Bierwirth P, Stockhorst U (2022) Role of noradrenergic arousal for fear extinction processes in rodents and humans. Neurobiol Learn Mem 194:107660. 10.1016/j.nlm.2022.107660 35870717

[B4] Binette AN, Totty MS, Maren S (2022) Sex differences in the immediate extinction deficit and renewal of extinguished fear in rats. PLoS One 17:e0264797. 10.1371/journal.pone.0264797 35687598 PMC9187087

[B5] Bloodgood DW, Sugam JA, Holmes A, Kash TL (2018) Fear extinction requires infralimbic cortex projections to the basolateral amygdala. Transl Psychiatry 8:60. 10.1038/s41398-018-0106-x 29507292 PMC5838104

[B6] Bukalo O, Pinard CR, Silverstein S, Brehm C, Hartley ND, Whittle N, Colacicco G, Busch E, Patel S, Singewald N, Holmes A (2015) Prefrontal inputs to the amygdala instruct fear extinction memory formation. Sci Adv 1:e1500251. 10.1126/sciadv.1500251 26504902 PMC4618669

[B7] Burgos-Robles A, Vidal-Gonzalez I, Santini E, Quirk GJ (2007) Consolidation of fear extinction requires NMDA receptor-dependent bursting in the ventromedial prefrontal cortex. Neuron 53:871–880. 17359921 10.1016/j.neuron.2007.02.021

[B8] Chang C, Maren S (2009) Early extinction after fear conditioning yields a context-independent and short-term suppression of conditional freezing in rats. Learn Mem 16:62–68. 10.1101/lm.1085009 19141467 PMC2632848

[B9] Chang C, Maren S (2011) Medial prefrontal cortex activation facilitates re-extinction of fear in rats. Learn Mem 18:221–225. 10.1101/lm.2070111 21430044 PMC3072771

[B10] Chang C, Berke JD, Maren S (2010) Single-unit activity in the medial prefrontal cortex during immediate and delayed extinction of fear in rats. PLoS One 5:e11971. 10.1371/journal.pone.0011971 20700483 PMC2916837

[B11] Chauveau F, Lange MD, Jüngling K, Lesting J, Seidenbecher T, Pape H-C (2012) Prevention of stress-impaired fear extinction through neuropeptide s action in the lateral amygdala. Neuropsychopharmacology 37:1588–1599. 10.1038/npp.2012.3 22298122 PMC3358750

[B12] Clemens AM, Lenschow C, Beed P, Li L, Sammons R, Naumann RK, Wang H, Schmitz D, Brecht M (2019) Estrus-cycle regulation of cortical inhibition. Curr Biol 29:605–615.e6. 10.1016/j.cub.2019.01.045 30744972

[B13] Courtin J, Chaudun F, Rozeske RR, Karalis N, Gonzalez-Campo C, Wurtz H, Abdi A, Baufreton J, Bienvenu TCM, Herry C (2014) Prefrontal parvalbumin interneurons shape neuronal activity to drive fear expression. Nature 505:92–96. 10.1038/nature12755 24256726

[B14] Cullinan WE, Herman JP, Battaglia DF, Akil H, Watson SJ (1995) Pattern and time course of immediate early gene expression in rat brain following acute stress. Neuroscience 64:477–505. 10.1016/0306-4522(94)00355-9 7700534

[B15] Cummings KA, Clem RL (2020) Prefrontal somatostatin interneurons encode fear memory. Nat Neurosci 23:61–74. 10.1038/s41593-019-0552-7 31844314 PMC6930333

[B16] Cummings KA, Lacagnina AF, Clem RL (2021) GABAergic microcircuitry of fear memory encoding. Neurobiol Learn Mem 184:107504. 10.1016/j.nlm.2021.107504 34425220 PMC8640988

[B17] Dilgen J, Tejeda HA, O'Donnell P (2013) Amygdala inputs drive feedforward inhibition in the medial prefrontal cortex. J Neurophysiol 110:221–229. 10.1152/jn.00531.2012 23657281 PMC3727030

[B18] Do-Monte FH, Manzano-Nieves G, Quiñones-Laracuente K, Ramos-Medina L, Quirk GJ (2015) Revisiting the role of infralimbic cortex in fear extinction with optogenetics. J Neurosci 35:3607–3615. 10.1523/JNEUROSCI.3137-14.2015 25716859 PMC4339362

[B19] Fitzgerald PJ, Giustino TF, Seemann JR, Maren S (2015) Noradrenergic blockade stabilizes prefrontal activity and enables fear extinction under stress. Proc Natl Acad Sci U S A 112:E3729–E3737. 10.1073/pnas.1500682112 26124100 PMC4507202

[B20] Floresco SB, Tse MT (2007) Dopaminergic regulation of inhibitory and excitatory transmission in the basolateral amygdala-prefrontal cortical pathway. J Neurosci 27:2045–2057. 10.1523/JNEUROSCI.5474-06.2007 17314300 PMC6673549

[B21] Giustino TF, Seemann JR, Acca GM, Goode TD, Fitzgerald PJ, Maren S (2017) β-Adrenoceptor blockade in the basolateral amygdala, but not the medial prefrontal cortex, rescues the immediate extinction deficit. Neuropsychopharmacology 42:2537–2544. 10.1038/npp.2017.89 28462941 PMC5686500

[B22] Giustino TF, Fitzgerald PJ, Ressler RL, Maren S (2019) Locus coeruleus toggles reciprocal prefrontal firing to reinstate fear. Proc Natl Acad Sci U S A 116:8570–8575. 10.1073/pnas.1814278116 30971490 PMC6486780

[B23] Giustino TF, Ramanathan KR, Totty MS, Miles OW, Maren S (2020) Locus coeruleus norepinephrine drives stress-induced increases in basolateral amygdala firing and impairs extinction learning. J Neurosci 40:907–916. 10.1523/JNEUROSCI.1092-19.2019 31801809 PMC6975297

[B24] Gruene TM, Roberts E, Thomas V, Ronzio A, Shansky RM (2015) Sex-specific neuroanatomical correlates of fear expression in prefrontal-amygdala circuits. Biol Psychiatry 78:186–193. 10.1016/j.biopsych.2014.11.014 25579850 PMC4449316

[B25] Izquierdo A, Wellman CL, Holmes A (2006) Brief uncontrollable stress causes dendritic retraction in infralimbic cortex and resistance to fear extinction in mice. J Neurosci 26:5733–5738. 10.1523/JNEUROSCI.0474-06.2006 16723530 PMC6675270

[B26] Joffe ME, et al. (2022) Acute restraint stress redirects prefrontal cortex circuit function through mGlu5 receptor plasticity on somatostatin-expressing interneurons. Neuron 110:1068–1083.e5. 10.1016/j.neuron.2021.12.027 35045338 PMC8930582

[B27] Kim SC, Jo YS, Kim IH, Kim H, Choi J-S (2010) Lack of medial prefrontal cortex activation underlies the immediate extinction deficit. J Neurosci 30:832–837. 10.1523/JNEUROSCI.4145-09.2010 20089891 PMC6633106

[B28] Kim W, Chung C (2021) Brain-wide cellular mapping of acute stress-induced activation in male and female mice. FASEB J 35:e22041. 10.1096/fj.202101287R 34780680

[B29] Knox D, George SA, Fitzpatrick CJ, Rabinak CA, Maren S, Liberzon I (2012) Single prolonged stress disrupts retention of extinguished fear in rats. Learn Mem 19:43–49. 10.1101/lm.024356.111 22240323 PMC3262971

[B30] Laurent V, Westbrook RF (2009) Inactivation of the infralimbic but not the prelimbic cortex impairs consolidation and retrieval of fear extinction. Learn Mem 16:520–529. 10.1101/lm.1474609 19706835

[B31] Marek R, Jin J, Goode TD, Giustino TF, Wang Q, Acca GM, Holehonnur R, Ploski JE, Fitzgerald PJ, Lynagh T, Lynch JW, Maren S, Sah P (2018) Hippocampus-driven feed-forward inhibition of the prefrontal cortex mediates relapse of extinguished fear. Nat Neurosci 21:384–392. 10.1038/s41593-018-0073-9 29403033 PMC5957529

[B32] Maren S (2022) Unrelenting fear under stress: neural circuits and mechanisms for the immediate extinction deficit. Front Syst Neurosci 16:888461. 10.3389/fnsys.2022.888461 35520882 PMC9062589

[B33] Maren S, Chang C (2006) Recent fear is resistant to extinction. Proc Natl Acad Sci U S A 103:18020–18025. 10.1073/pnas.0608398103 17090669 PMC1693865

[B34] Maren S, Holmes A (2016) Stress and fear extinction. Neuropsychopharmacology 41:58–79. 10.1038/npp.2015.180 26105142 PMC4677122

[B35] Maroun M, Ioannides PJ, Bergman KL, Kavushansky A, Holmes A, Wellman CL (2013) Fear extinction deficits following acute stress associate with increased spine density and dendritic retraction in basolateral amygdala neurons. Eur J Neurosci 38:2611–2620. 10.1111/ejn.12259 23714419 PMC3773716

[B36] McGarry LM, Carter AG (2016) Inhibitory gating of basolateral amygdala inputs to the prefrontal cortex. J Neurosci 36:9391–9406. 10.1523/JNEUROSCI.0874-16.2016 27605614 PMC5013187

[B37] Milad MR, Quirk GJ (2002) Neurons in medial prefrontal cortex signal memory for fear extinction. Nature 420:70–74. 10.1038/nature01138 12422216

[B38] Milad MR, Vidal-Gonzalez I, Quirk GJ (2004) Electrical stimulation of medial prefrontal cortex reduces conditioned fear in a temporally specific manner. Behav Neurosci 118:389–394. 10.1037/0735-7044.118.2.389 15113265

[B39] Moench KM, Breach MR, Wellman CL (2020) Prior stress followed by a novel stress challenge results in sex-specific deficits in behavioral flexibility and changes in gene expression in rat medial prefrontal cortex. Horm Behav 117:104615. 10.1016/j.yhbeh.2019.104615 31634476 PMC6980662

[B40] Nawreen N, Cotella EM, Morano R, Mahbod P, Dalal KS, Fitzgerald M, Martelle S, Packard BA, Franco-Villanueva A, Moloney RD, Herman JP (2020) Chemogenetic inhibition of infralimbic prefrontal cortex gabaergic parvalbumin interneurons attenuates the impact of chronic stress in male mice. eNeuro 7:ENEURO.0423-19.2020. 10.1523/ENEURO.0423-19.2020 PMC7598911 33055196

[B41] Page CE, Coutellier L (2019) Prefrontal excitatory/inhibitory balance in stress and emotional disorders: evidence for over-inhibition. Neurosci Biobehav Rev 105:39–51. 10.1016/j.neubiorev.2019.07.024 31377218

[B42] Page CE, Shepard R, Heslin K, Coutellier L (2019) Prefrontal parvalbumin cells are sensitive to stress and mediate anxiety-related behaviors in female mice. Sci Rep 9:19772. 10.1038/s41598-019-56424-9 31875035 PMC6930291

[B43] Ramanathan KR, Jin J, Giustino TF, Payne MR, Maren S (2018) Prefrontal projections to the thalamic nucleus reuniens mediate fear extinction. Nat Commun 9:4527. 10.1038/s41467-018-06970-z 30375397 PMC6207683

[B44] Senn V, Wolff SBE, Herry C, Grenier F, Ehrlich I, Gründemann J, Fadok JP, Müller C, Letzkus JJ, Lüthi A (2014) Long-range connectivity defines behavioral specificity of amygdala neurons. Neuron 81:428–437. 10.1016/j.neuron.2013.11.006 24462103

[B45] Shansky RM, Hamo C, Hof PR, Lou W, McEwen BS, Morrison JH (2010) Estrogen promotes stress sensitivity in a prefrontal cortex–amygdala pathway. Cereb Cortex 20:2560–2567. 10.1093/cercor/bhq003 20139149 PMC2951843

[B46] Sierra-Mercado D, Padilla-Coreano N, Quirk GJ (2011) Dissociable roles of prelimbic and infralimbic cortices, ventral hippocampus, and basolateral amygdala in the expression and extinction of conditioned fear. Neuropsychopharmacology 36:529–538. 10.1038/npp.2010.184 20962768 PMC3005957

[B47] Sood A, Chaudhari K, Vaidya VA (2018) Acute stress evokes sexually dimorphic, stressor-specific patterns of neural activation across multiple limbic brain regions in adult rats. Stress 21:136–150. 10.1080/10253890.2017.1422488 29316846

[B48] Swanson LW (2004) Brain maps: structure of the rat brain, Ed 3. Amsterdam: Elsevier.

[B49] Vormstein-Schneider D, et al. (2020) Viral manipulation of functionally distinct interneurons in mice, non-human primates and humans. Nat Neurosci 23:1629–1636. 10.1038/s41593-020-0692-9 32807948 PMC8015416

[B50] Wilber AA, Walker AG, Southwood CJ, Farrell MR, Lin GL, Rebec GV, Wellman CL (2011) Chronic stress alters neural activity in medial prefrontal cortex during retrieval of extinction. Neuroscience 174:115–131. 10.1016/j.neuroscience.2010.10.070 21044660 PMC3020264

[B51] Woodward EM, Coutellier L (2021) Age- and sex-specific effects of stress on parvalbumin interneurons in preclinical models: relevance to sex differences in clinical neuropsychiatric and neurodevelopmental disorders. Neurosci Biobehav Rev 131:1228–1242. 10.1016/j.neubiorev.2021.10.031 34718048 PMC8642301

[B52] Yang S-S, Mack NR, Shu Y, Gao W-J (2021) Prefrontal GABAergic interneurons gate long-range afferents to regulate prefrontal cortex-associated complex behaviors. Front Neural Circuits 15:716408. 10.3389/fncir.2021.716408 34322002 PMC8313241

